# Engineered variants of the Ras effector protein RASSF5 (NORE1A) promote anticancer activities in lung adenocarcinoma

**DOI:** 10.1016/j.jbc.2021.101353

**Published:** 2021-10-27

**Authors:** Anamika Singh, Ariel Erijman, Ashish Noronha, Hemant Kumar, Yoav Peleg, Yosef Yarden, Julia M. Shifman

**Affiliations:** 1 Department of Biological Chemistry, The Alexander Silberman Institute of Life Sciences, The Hebrew University of Jerusalem, Jerusalem, Israel; 2 Department of Biological Regulation, Weizmann Institute of Science, Rehovot, Israel; 3 Life Sciences Core Facilities (LSCF) Structural Proteomics Unit (SPU), Weizmann Institute of Science, Rehovot, Israel

**Keywords:** Ras oncogene, Ras effector protein, RASSF5, Nore1A, Ras inhibitors, protein engineering, protein-protein interactions, APC, allophycocyanin, ERK, extracellular signal-regulated kinase, FACS, fluorescence-activated cell sorting, FITC, fluorescein isothiocyanate, GEF, guanine nucleotide exchange factor, MEK, mitogen-activated protein kinase, PLC, phospholipase C, RASSF5, Ras association domain family 5, RBD, RAS-binding domain, SARAH, Salvador-RASSF-Hippo, SOS, son of sevenless, SPR, surface plasmon resonance, YSD, yeast surface display

## Abstract

Within the superfamily of small GTPases, Ras appears to be the master regulator of such processes as cell cycle progression, cell division, and apoptosis. Several oncogenic Ras mutations at amino acid positions 12, 13, and 61 have been identified that lose their ability to hydrolyze GTP, giving rise to constitutive signaling and eventually development of cancer. While disruption of the Ras/effector interface is an attractive strategy for drug design to prevent this constitutive activity, inhibition of this interaction using small molecules is impractical due to the absence of a cavity to which such molecules could bind. However, proteins and especially natural Ras effectors that bind to the Ras/effector interface with high affinity could disrupt Ras/effector interactions and abolish procancer pathways initiated by Ras oncogene. Using a combination of computational design and *in vitro* evolution, we engineered high-affinity Ras-binding proteins starting from a natural Ras effector, RASSF5 (NORE1A), which is encoded by a tumor suppressor gene. Unlike previously reported Ras oncogene inhibitors, the proteins we designed not only inhibit Ras-regulated procancer pathways, but also stimulate anticancer pathways initiated by RASSF5. We show that upon introduction into A549 lung carcinoma cells, the engineered RASSF5 mutants decreased cell viability and mobility to a significantly greater extent than WT RASSF5. In addition, these mutant proteins induce cellular senescence by increasing acetylation and decreasing phosphorylation of p53. In conclusion, engineered RASSF5 variants provide an attractive therapeutic strategy able to oppose cancer development by means of inhibiting of procancer pathways and stimulating anticancer processes.

Cellular signal transduction is mediated by protein/protein interactions leading to spatial and temporal organization of cellular constituents and to specific up- and downregulation of enzymatic activities. Here, the members of the superfamily of small GTP-binding proteins play pivotal roles as they stand in the center of diverse biological processes. Among small GTP-binding proteins, Ras is the master regulator of cell cycle progression, cell migration, adhesion, differentiation, and apoptosis (1, 2). Ras cycles between an active, GTP-bound state (Ras-GTP), and an inactive, GDP-bound state (Ras-GDP) (3, 4). Switching to the active state of Ras is promoted by binding of a guanine nucleotide exchange factor (GEF) that increases the nucleotide dissociation rate of Ras and promotes loading with GTP, which is present in excess over GDP in the intracellular environment. Switching to the inactive state occurs upon GTP hydrolysis to GDP and is increased by several orders of magnitude on binding of a GTPase activating protein GAP (5).

Structurally, the GDP- and the GTP-bound Ras forms exhibit only small differences in two regions, approximately ten amino acids each: a loop that directly contacts the effector protein (switch I) and a loop and a helix that are close to the effector-binding site (switch II) (Fig. 1*A*). In spite of these small structural changes, Ras affinity to its effectors varies depending on the bound nucleotide. In the GTP-bound state, Ras can bind to various effectors with physiologically relevant binding affinities ranging from 0.1 μM to 3 μM (6, 7). In the GDP-bound state, the affinity of Ras to effectors is substantially reduced (8).

**Figure 1:**
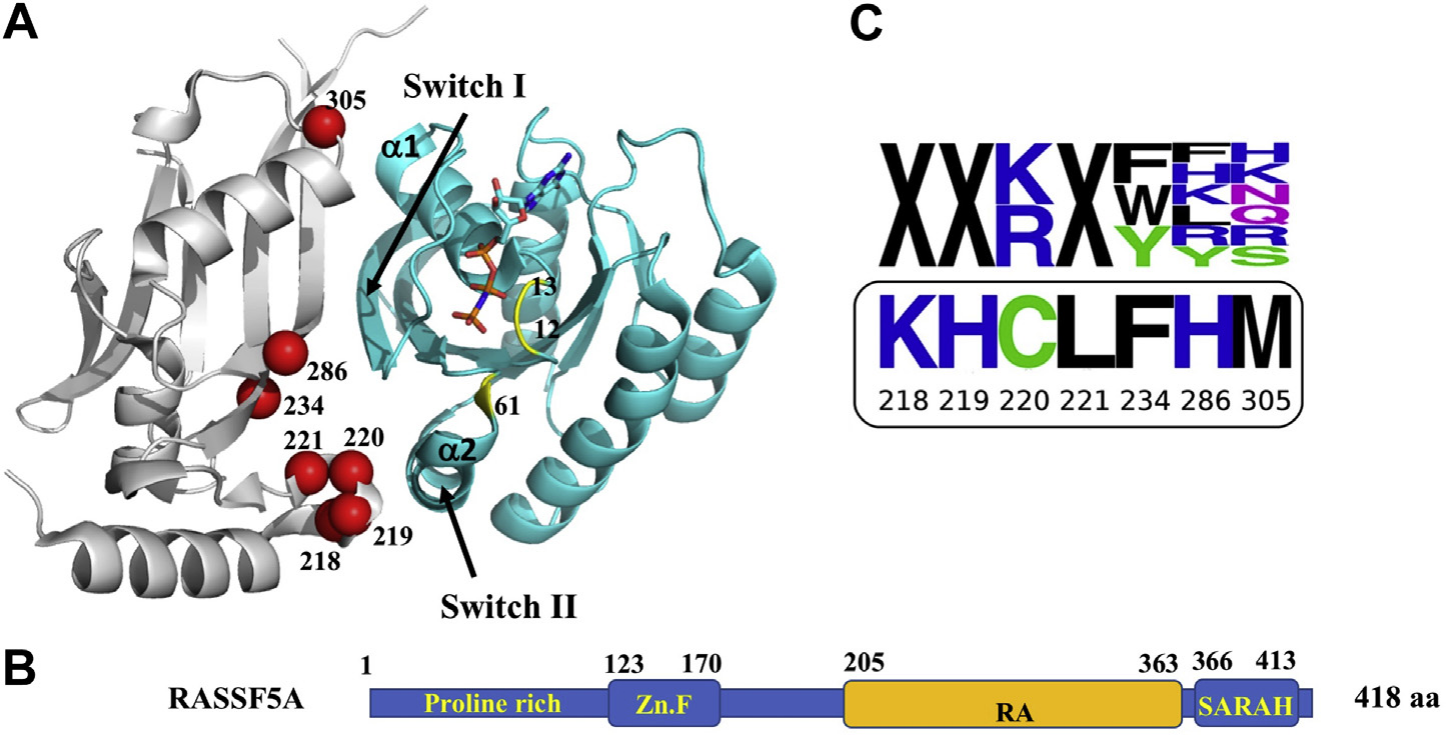
**RASSF5-Ras** a**ssociation domain (RA) library design and schematic representation of the RASSF5 gene.***A*, structure of RASSF5 (*gray*) in complex with Ras-GTP(*cyan*) (PDB ID 3DDC). Switch I and II are indicated by arrows and the site of oncogenic mutations is colored in *yellow*. Positions on RASSF5 that were selected for randomization are shown as *red spheres*. *B*, schematic representation of RASSF5 full gene including largely unfolded N-terminal domain with a proline-rich region and a zink finger (Zn.F) domain, RA and Salvador-RASSF-Hippo (SARAH) domain. *C*, Weblogos of the RASSF5 designed library (*upper panel*) and wild-type sequence (*bottom*). X denotes randomization to all 20 amino acids with an NNS codon.

Multiple Ras effectors have been identified, including Raf protein kinase (9) that is involved in cell proliferation and differentiation, Ral guanine nucleotide dissociation stimulator (10) that participates in RAS-dependent tumorigenesis, phosphoinositide 3-kinase that stimulates cell survival (11), phospholipase C epsilon (PLC) that senses and mediates cross talk between heterotrimeric and small GTPase signaling pathways (12) and the RASSF family of effectors promoting cell death and senescence (13, 14, 15). Although Ras effectors exhibit substantial differences in sequence and function, they all possess an 80 to 100-amino-acid RAS-Association (RA) domain (also called RAS Binding Domain, RBD), which exhibits a ubiquitin-like fold (6). Moreover, all the effectors bind to the same binding site on Ras, forming an antiparallel intermolecular β-sheet between β2 of the RA domain of the effector and β2 of Ras (7) (Fig. 1*A*). The contacts between Ras and a typical RA domain are largely polar, including several favorable hydrogen bond and salt bridge interactions across the binding interface (16).

Several point mutations in Ras are associated with cancer. Roughly 30% of human tumors contain Ras mutations with the highest frequency occurring in pancreatic, colorectal, and lung carcinomas (17, 18, 19). Most common oncogenic mutations are located at three positions on Ras: 12, 13, and 61, all situated near the nucleotide-binding site (Fig. 1*A*). The oncogenic Ras mutants are not able to convert GTP to GDP, hence are locked in the “on” state where they constantly activate cell cycle progression and division. Such activation, together with mutations in other oncogenes or tumor suppressor genes, eventually transforms the cells into cancerous cells (20).

While the important role of Ras in cancer has been long established, Ras has been considered an undruggable target, mostly due to the fact that the Ras/effector interface is flat and contains no cavity able to encore a small molecule (21). Yet, in recent years we witness a resurgence of studies that report inhibitors of Ras-driven oncogenesis using a number of different strategies. In one approach, a small-molecule inhibitor of the Ras G12C mutant was developed to bind irreversibly through the mutated cysteine (22). In two different studies, small-molecule inhibitors have been developed to bind to and disrupt the interface between Ras and its nucleotide exchange factor son of sevenless (SOS), thus preventing nucleotide exchange and subsequent effector binding (23, 24). Moreover, a number of protein-based Ras inhibitors have been developed that have a potential to bind oncogenic Ras with high affinity and high selectivity. Among them is a monobody that disrupts Ras dimerization interface, which is crucial for signaling (25), a single immunoglobulin VH domain that recognizes the activated Ras with subnanomolar affinity (26), inhibitors based on fibronectin domain that recognize selectively the Ras-G12V mutant (27), inhibitors based on the Ras effector Raf (28) or on DARPins (29). The use of these engineered proteins in cellular settings and animal models revealed that Ras inhibition is a promising strategy to counteract Ras-driven oncogenesis (28, 30, 31).

In this study we present a high-affinity inhibitor of Ras based on the RA domain of a natural Ras effector, Ras Association Domain Family 5 (RASSF5) (Fig. 1*B*). RASSF5 belongs to a large family of cancer suppressor effectors that are inactivated by promoter hypermethylation in numerous cancer cell lines and primary cancers (32, 33). While catalytically inactive, RASSF5 serves as an adaptor protein that links Ras signaling to proapoptotic and prosenescence pathways (34). Like other Ras effectors, RASSF5 uses the RA domain to interact with switches I and II on Ras. However, it also interacts with Ras helixes α1 and α2, exhibiting larger binding interface and longer association half-life compared to other known Ras effectors (7, 35). Besides the RA domain, RASSF5 contains an intrinsically unfolded N-terminal domain containing a proline-rich region that can bind SH3 domains and a helical Salvador-RASSF-Hippo (SARAH) domain (Fig. 1*B*). The latter is used for RASSF5 homodimerization (36) and could also heterodimerize with the SARAH domain of the Hippo kinases MST1/2, to promote apoptosis (37, 38, 39). In addition, RASSF5 was shown to activate several tumor suppressors, including p53 and the retinoblastoma (Rb) protein, through posttranslational modifications, thereby promoting senescence (40, 41). The natural role of RASSF5 as a cancer suppressor that induces senescence and proapoptotic pathways makes it an attractive candidate for inhibiting Ras-driven cancers. Using a powerful combination of computational protein design and directed evolution (42), we engineered RASSF5 RA domain variants that enhance its binding affinity to Ras-GTP and Ras-GDP and demonstrate that these variants inhibit Ras-associated cancer progression in lung cancer cells.

## Results

### Design of a focused RASSF5 library

RASSF5 utilizes 15 residues to interact with Ras. In principle, we could randomize all of these residues to select the best binders to Ras through directed evolution. However, such randomization would produce a library of ∼10^19^ variants, which could not be fully explored experimentally through the Yeast Surface Display (YSD) technology. Thus, we decided to design a focused library of RASSF5 mutants to limit randomization to only most promising positions and amino acids. For this purpose, we first performed computational saturation mutagenesis of the RASSF5/Ras-GTP complex (43), where all the binding interface residues on RASSF5 were mutated to 17 amino acids. Mutations to Pro, Gly, and Cys were excluded from consideration due to their potential undesired impact on the structure of the protein. During the calculation, individual mutations were separately introduced, one mutation at a time, into the RASSF5 interface, while the surrounding residues were repacked and the change in binding free energy (ΔΔG_bind_) to Ras due to mutation was computed. Our analysis showed that at several positions (*e.g.*, at positions 220, 277, 286, 304, 305), at least one substitution was predicted to substantially improve ΔΔG_bind_ to Ras (Fig. S1).

We next selected for randomization the RASSF5 residues that were either predicted to have suboptimal interactions with Ras or were in contact with Ras switches, hence could sense small structural changes due to GTP hydrolysis. These included three groups (Fig. 1*A*):

(i) residues 218 to 221 comprising a loop that is in contact with switch II on Ras including residues 218, 220, 221 that directly contact Ras and residue 219 that interacts with the above residues and defines the loop conformation.
(ii) residue 286 with a high potential for improvement of intermolecular interactions and a neighboring residue 234. Both positions are interacting with switch I of Ras and are coupled to each other.
(iii) residue 305 that is in close proximity to switch I of Ras and also shows good potential for affinity improvement.

For experimental convenience, we sought to design a single library that contained high-affinity binders to both Ras-GTP and Ras-GDP and at the same time was small enough to be fully explored in the YSD setup. We thus performed two calculations where we simultaneously optimized all seven RASSF5 positions while it was interacting with Ras-GTP and separately with Ras-GDP.

A lysine was predicted at position 220 in both calculations, and we decided to incorporate a lysine and an arginine at this position in the library. At positions 218, 219, and 221 we did not see a clear amino acid preference for only one nucleotide-bound Ras state, and we decided to randomize these positions with all 20 amino acids. Glutamine and lysine were predicted at positions 234 and 286, respectively. To reduce the probability of unfolding of the RASSF5 mutants, we allowed only aromatic residues at position 234 (as phenylalanine is observed in the WT protein) and only positively charged and aromatic residues at position 286, which were predicted to improve intermolecular interactions. Finally, the last residue, M305, is in the proximity of D30 and E31 on Ras. A lysine at this position could introduce favorable electrostatic and hydrogen bond interactions with these residues. In the library, we included positive and polar amino acids at position 305. We thus designed a single RASSF5 library containing variants with improved binding to both Ras-GTP and Ras-GDP, while limiting its size to 1.7 × 10^6^ variants to allow for full assessment of all variants with the YSD technology (Fig. 1*C*).

### Selection of RASSF5 mutants with improved binding to Ras-GTP and Ras-GDP

**Figure 2:**
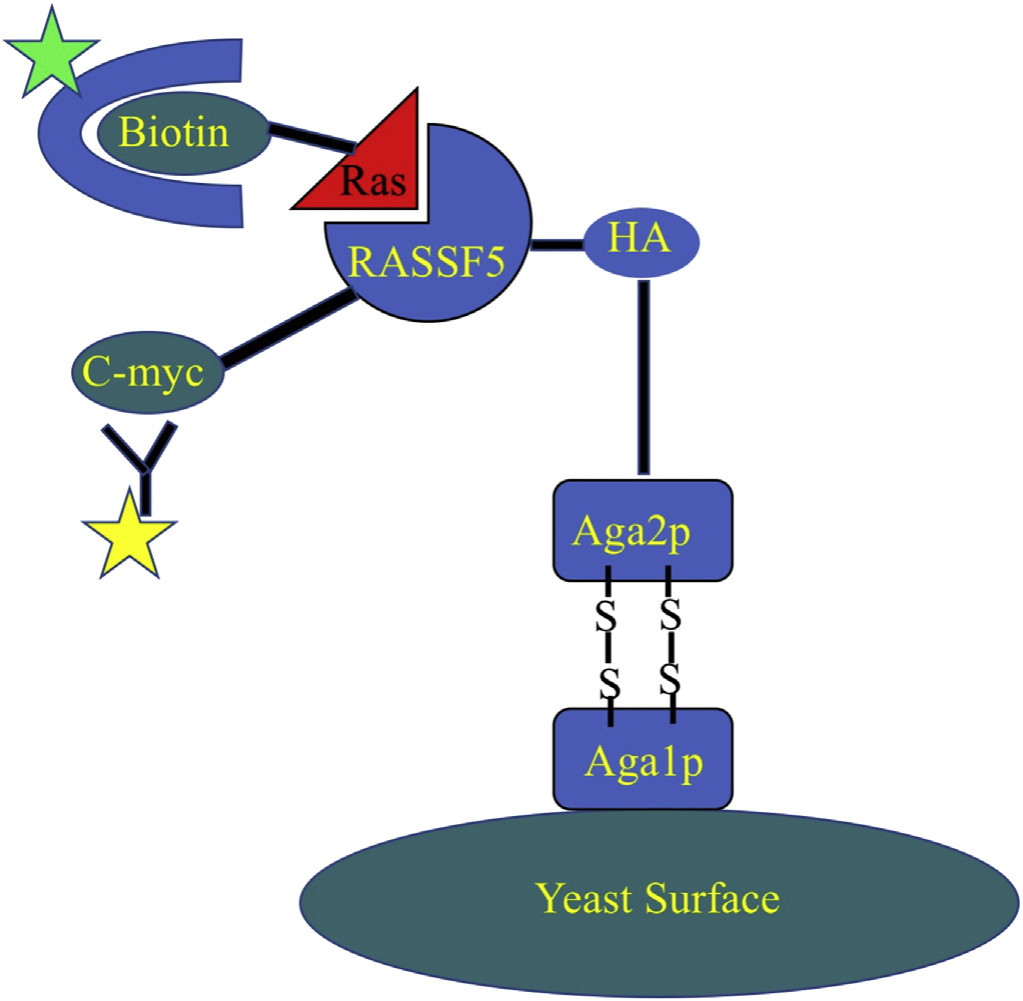
**Schematic representation of the yeast surface display setup in this work.** RASSF5 library was expressed on the surface of the yeast cells coupled to a c-myc tag at the C-terminal and incubated with biotinylated-Ras (either in the GTP or in the GDP form). Fluorescein isothiocyanate (FITC)-labeled anti-myc antibody (*yellow*) and Allophycocyanin (APC)-labeled streptavidin (*green*) were used to detect RASSF5 expression and binding to Ras, respectively.

**Figure 3:**
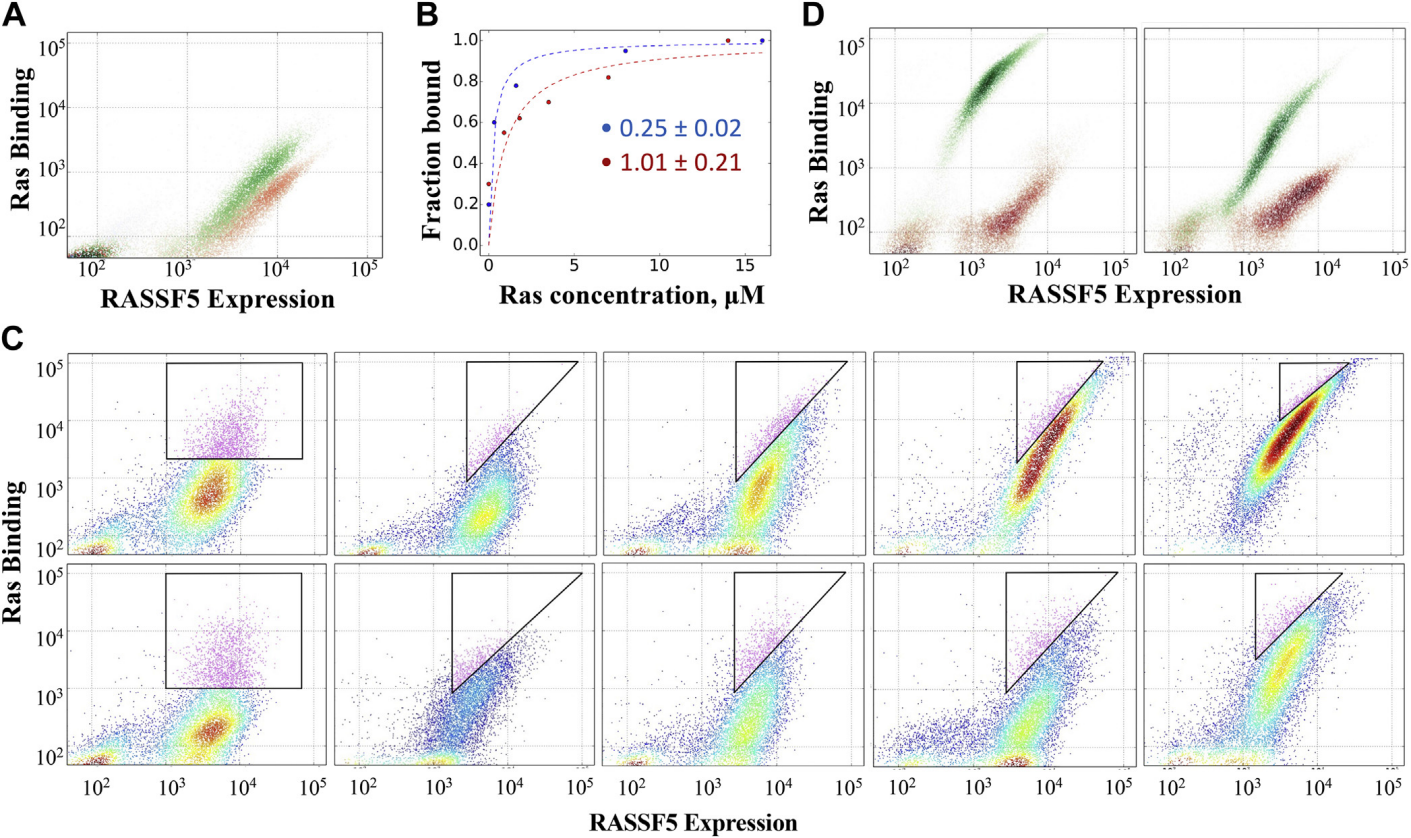
**Fluorescence-activated cell sorting (FACS)analysis and sorting of RASSF5 mutants binding to Ras-GTP and Ras-GDP.***A*, Ras-GTP (*green* – 2.5 μM) and Ras-GDP (*red* – 5.0 μM) binding to RASSF5 wild type expressed on the yeast cell surface. *B*, titrations of Ras-GTP (*blue*) and Ras-GDP (*red*) into RASSF5 wild type expressed on the yeast cell surface. Data from density plots was used to measure apparent Kd of RASSF5 WT and Ras-GTP and Ras-GDP as indicated on the plot. *C*, sorting of the RASSF5 mutant library when binding to Ras-GTP (*upper panel*) and Ras-GDP (*lower panel*). For both sorts, the first panel to the left, shows presorting of the RASSF5 mutants. Subsequent four rounds of selection of best binders are depicted from *left to right*. Triangles represent the 3 to 5% of best binders selected in each round. *D*, Ras-GTP at 0.2 μM (*left panel*) and Ras-GDP at 2 μM (*right panel*) binding an engineered RASSF5 mutant selected form the library (*green*) compared with RASSF5 wild type (*red*). In all experiments FITC is used to monitor RASSF5 variant expression while APC is used to monitor Ras binding.

The designed library of RASSF5 mutants was constructed (see Experimental procedures) and incorporated into a pCTCON2 vector compatible with the YSD platform (Fig. 2). Sequencing of ten clones from the initial library confirmed that all of the desired positions were randomized to the desired codons. The library of RASSF5 mutants was expressed on the surface of yeast cells and labeled with fluorescein isothiocyanate (FITC), to monitor protein expression. Ras, either in the GTP or the GDP form, labeled with allophycocyanin (APC) was added to the yeast cells, allowing us to monitor binding. To verify expression and correct folding of RASSF5 on yeast, we first analyzed fluorescence-activated cell sorting (FACS) signals from WT RASSF5 binding to Ras-GTP. Figure 3*A* shows that interaction between RASSF5 WT and Ras-GTP and Ras-GDP could be detected in the YSD setup, while no binding was observed for the negative control in which RASSF5 was not expressed (data not shown). Next, we titrated RASSF5 WT with Ras-GTP and Ras-GDP and determined the apparent K_D_s to be 0.25 ± 0.02 and 1.01 ± 0.21 μM for Ras-GTP and Ras-GDP, respectively (Fig. 3*B*). In the next step, we expressed the RASSF5 mutant library and sorted it for binding to Ras-GTP and Ras-GDP in two separate experiments (Fig. 3*C*). To eliminate the RASSF5 mutants that were not well expressed on the yeast surface, we presorted the library by collecting the top 5% of the population with the highest expression and binding signals (Fig. 3*C*, first panel from the left). We subsequently sorted the library four more times, each time collecting populations of RASSF5 variants that exhibited increased binding to either Ras-GTP or Ras-GDP (Fig. 3*C*, top and bottom panels, respectively). The concentrations of Ras-GTP and Ras-GDP in solution were progressively decreased with each selection round to collect only high-affinity clones. After four rounds of selection, several mutants from the RASSF5 library were expressed separately on the yeast surface and their binding to both isomers of Ras was compared using YSD (Fig. 3*D*). As expected, engineered RASSF5 mutants yielded considerably higher signal for binding to Ras, as compared with WT RASSF5, and displayed a spectrum of binding specificities toward the two different states of Ras.

### Sequence analysis

**Figure 4:**
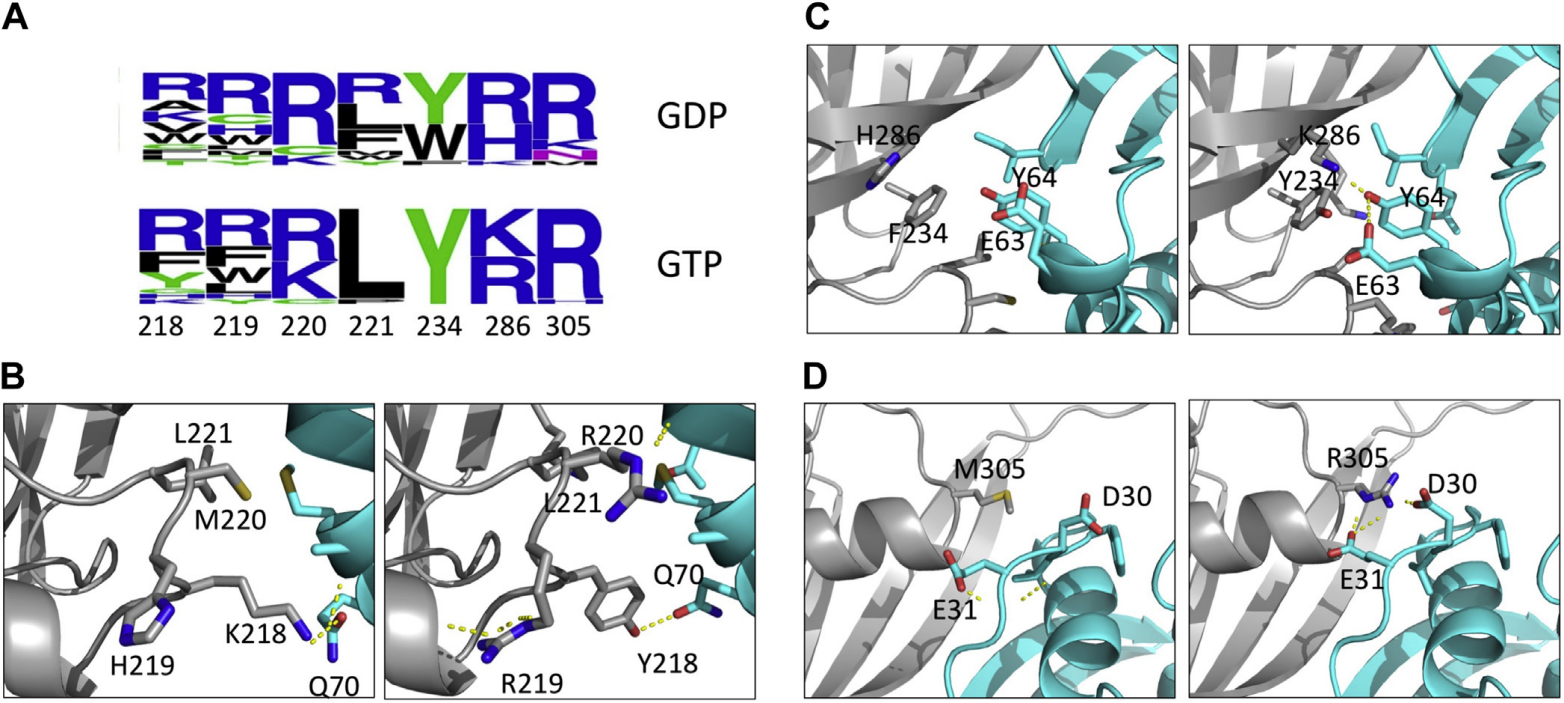
**Sequence profiling of the RASSF5 mutants.***A*, WebLogo representation of the RASSF5 sequences selected for binding to Ras-GDP (*top*) and for Ras-GTP (*bottom*). *B*–*D*, WT (*left*) and engineered (*right*) RASSF5 mutants interacting with Ras-GTP. RASSF5 is in *gray* and Ras is in *cyan*. WT interactions are shown according to the PDB structure 3DDC. With 3 introduced mutations to revert the sequences of both proteins back to the WT sequences: D302K on RASSF5 and E30D/K31E on Ras. Engineered interactions were modeled using the ORBIT software. Residues participating in intermolecular interactions are shown as *sticks*, hydrogen bonds are shown as *yellow dotted lines*.

Following four rounds of selection, we sequenced 20 RASSF5 variants from the Ras-GTP and Ras-GDP selections and compared their sequences (Fig. 4*A* and Table S1). The results showed that sequences with increased affinity to both Ras-GTP and Ras-GDP were dominated by positively charged residues at most of the positions, excluding positions 221 and 234, which were occupied by hydrophobic amino acids. However, each selected RASSF5 sequence carried at most four charged residues at a time, increasing the total charge of RASSF5 mutant by three at maximum compared with WT RASSF5. High frequency of arginines and lysines among the selected RASSF5 binders was not surprising due to two reasons. First, Ras-binding interface is rich in negatively charged residues (such as D30, E31, E37, D38, E63) and potential hydrogen bond acceptors (such as Q70, Q25, Y40, and Y64) that could partake in favorable electrostatic and hydrogen bond interactions with lysines and arginines on RASSF5. Second, lysines and arginines are found frequently in protein–protein interfaces (44) due to their ability to participate in both hydrophobic and polar interactions, with arginines being among the most abundant residues in binding hotspots (45). RASSF5 mutants selected for Ras-GTP and Ras-GDP were generally similar, exhibiting slight differences in their sequence consensus (Fig. 4*A*). To analyze the effect of the introduced mutations in the engineered RASSF5 mutants on binding to Ras, we modeled the structures of several RASSF5 mutant/Ras complexes (Fig. 4, *B*–*D*).

Our modeling shows that a positively charged residue at position 219 could participate in C-capping of a helix on RASSF5, stabilizing the unbound protein, while Tyr at position 218 could create a new intermolecular hydrogen bond with Gln 70 (Fig. 4*B*). At position 220, a newly introduced arginine could form a hydrogen bond to the backbone of residue 64 on Ras. Interestingly, position 221 remains a WT leucine in 100% of cases for the Ras-GTP selection, but it is replaced by an arginine or a hydrophobic residue in the Ras-GDP selection. On the contrary, tyrosine at position 234 is the most selected amino acid, reaching nearly 100% for GTP-Ras-selected clones and about 50% in the GDP-selected clones, with tryptophan being another option. Both mutations are predicted to improve packing between the two proteins. A positively charged residue at position 286 is selected; this residue is predicted to create a hydrogen bond network with E63 and Y64 on Ras (Fig. 4*C*). At position 305, WT methionine is substituted by an arginine that could create salt bridges with D30 and D31 on Ras (Fig. 4*D*).

### Binding affinity measurements

We next expressed and purified RASSF5 WT and six representative mutants, three obtained in the Ras-GTP selection (T1, T2, T3) and three obtained in the Ras-GDP selection (D1, D2, D3; see Table 1 for sequences). We measured binding affinities of RASSF5 mutants interacting with Ras-GTP and Ras-GDP using surface plasmon resonance (SPR). In these experiments, Ras was immobilized on a streptavidin chip *via* biotin and WT RASSF5, and mutants were flown in solution. We first measured binding between RASSF5 WT and the two Ras isoforms, which yielded K_D_ values of 2.6 and 5.1 μM for Ras-GTP and Ras-GDP, respectively (Table 1 and Figs. S2 and S3). The K_D_ measured with SPR for Ras-GTP/WT RASSF5 interaction was weaker than that observed for the same interaction using the GDI assay (46) however, is similar to that measured by ITC at same buffer conditions (15). While SPR likely gives us an underestimation of the binding strength for this interaction *in vivo*, we found this method reliable in comparing K_D_ values for various mutants of RASSF5 to Ras as such experiments are performed at exact same conditions. Using SPR, we measured K_D_ values for six expressed RASSF5 mutants to Ras-GTP and Ras-GDP (Table 1). Our results showed that the listed mutants exhibit up to approximately sevenfold enhancement in binding affinity to Ras by SPR, with K_D_s varying in the 0.4 to 1.1 μM and the 0.6 to 3.0 μM range for binding to Ras-GTP and Ras-GDP, respectively. K_D_ to Ras was generally improved for all mutants for the both Ras-GTP and Ras-GDP states, with all engineered mutants exhibiting higher affinity for Ras-GTP compared with Ras-GDP.

**Table 1:** Binding rates and affinities of RASSF5 variants for Ras-GTP and Ras-GDP

RASFF5 mutant		Ras-GTP	Ras-GDP
		K_d_, μM	K_d_,μM
WT	KHCLFHM^a^	2.6 ± 0.3	5.1 ± 0.4
T1	RLRLYKR	0.56 ± 0.08	-b
T2	YRRLYKR	0.53 ± 0.11	3.0 ± 0.5
T3	WSRLYKR	1.0 ± 0.15	1.6 ± 0.3
D1	NIKFYKR	0.41 ± 0.06	0.84 ± 0.13
D2	RTRHWKR	0.93 ± 0.14	1.6 ± 0.3
D3	FRKFWHK	1.1 ± 0.11	1.6 ± 0.2

a Only the sequence at randomized positions (218, 219, 220, 221, 234, 286, 305) is given.

b The data for this mutant is not available.

### Engineered RASSF5 variants inhibit Ras-associated cancer processes in lung cancer A549 cells

We next set out to test the activity of the engineered RASSF5 variants in cancer cells. For this, we selected the A549 human lung adenocarcinoma cell line for transfection since this line overexpresses an oncogenic KRas mutant (G12S) but expresses no endogenous RASSF5, thus allowing us to introduce our engineered mutants on a null background. While our mutants were initially optimized for HRas binding, they should exhibit similar affinities to KRas since the two Ras isoforms are identical in the effector binding interface region and exhibit similar K_D_ values to WT RASSF5 (47). For each RASSF5 mutant, we prepared two constructs, one containing only the RA domain with the engineered mutations incorporated, and the other containing the engineered RA domain followed by a SARAH domain, a C-terminal helical extension of 47 amino acids. We have attempted to perform stable cellular transfection of our RASSF5 constructs into A549 cells; however, cells transfected with our constructs were not viable in contrast to cells transfected with an empty vector (EV) control. This result could be due to cellular senescence and/or apoptosis elicited by the RASSF5 mutants or due to the strong inhibitory effect of RASSF5 mutants on the Ras/MAPK pathways that is crucial for growth of common cells lines (30). Thus, we have turned to transient transfection and transfected 12 engineered constructs and two WT RASSF5 into A549 cells and measured their effects on various cancer-related pathways. Transfection of EV was used as a negative control in all experiments.

**Figure 5:**
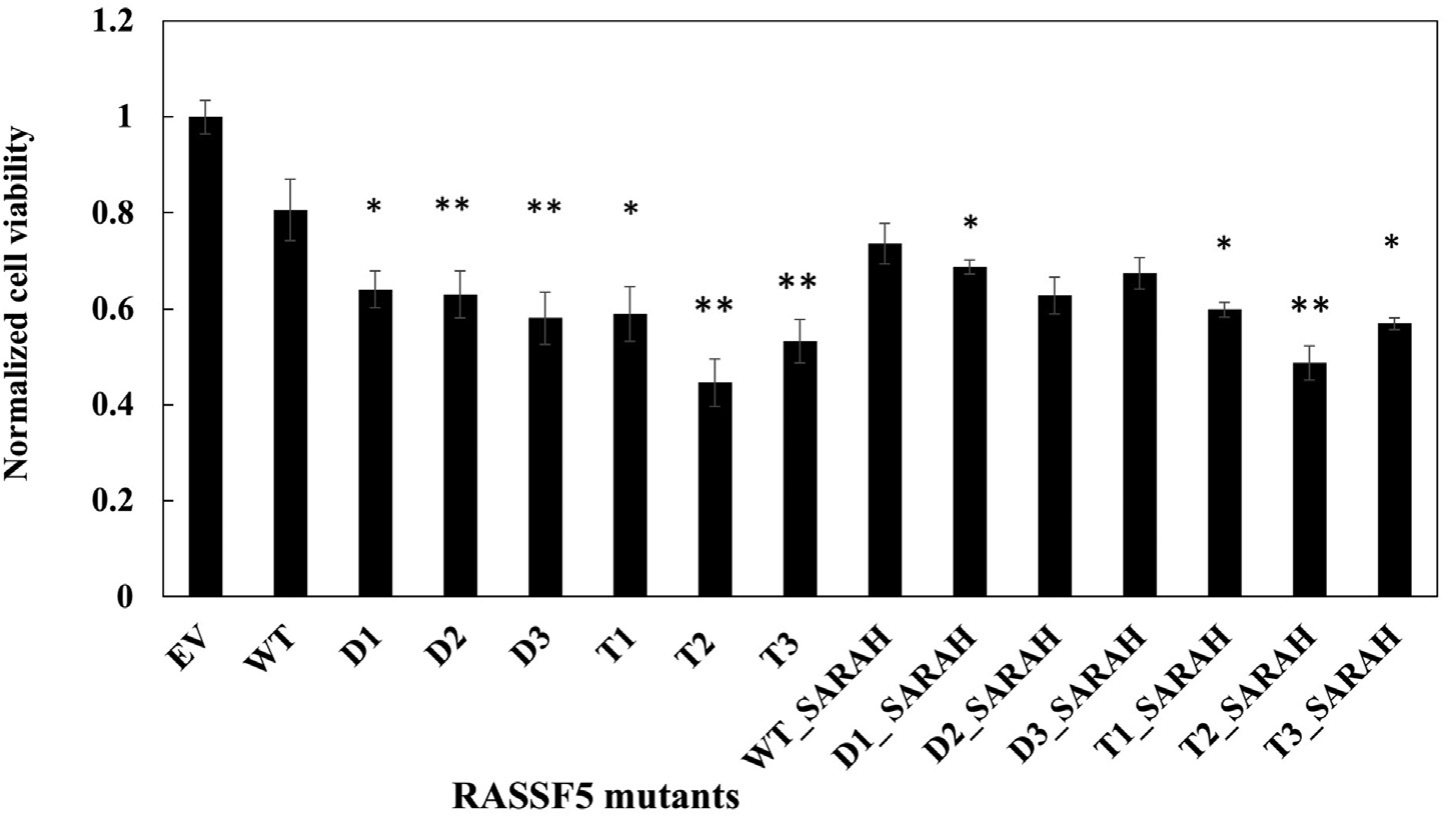
**EffectofRASSF5 mutants on metabolic activity of A549 cells.** A549 cells were transiently transfected with WT RASSF5 and mutants. Cell viability was measured using MTT assay at 48 h post transfection, quantified spectrophotometrically at 590 nm, and normalized to the value of Empty Vector (EV). The results represent the average of four independent experiments shown as mean ± SD. The level of significance is indicated by ∗ for *p* < 0.05 and ∗∗ for *p* < 0.01 *versus* WT RASSF5 in the paired one-tailed *t* test.

First, we assessed the ability of RASSF5 mutants to affect cell viability using the MTT assay, which is based on measuring cell metabolic activity, in particular activity of NAD(P)H-dependent cellular oxidoreductase enzymes (48). In such an assay, reduction of oxidoreductase substrate MTT is observed as an appearance of absorbance at 570 nm for the cells transfected with the engineered RASSF5 mutants. Our results showed that all engineered RASSF5 mutants inhibited cell viability and were more potent inhibitors compared with WT RASSF5 (Fig. 5). While WT RASSF5 RA inhibited cell viability by about 20%, the mutants showed 30 to 50% inhibition with the T2 mutant being the most potent inhibitor. Figure 5 shows that we observed no difference in inhibition by the RA or RA + SARAH constructs, revealing that the SARAH domain interactions are not important for cell viability.

**Figure 6:**
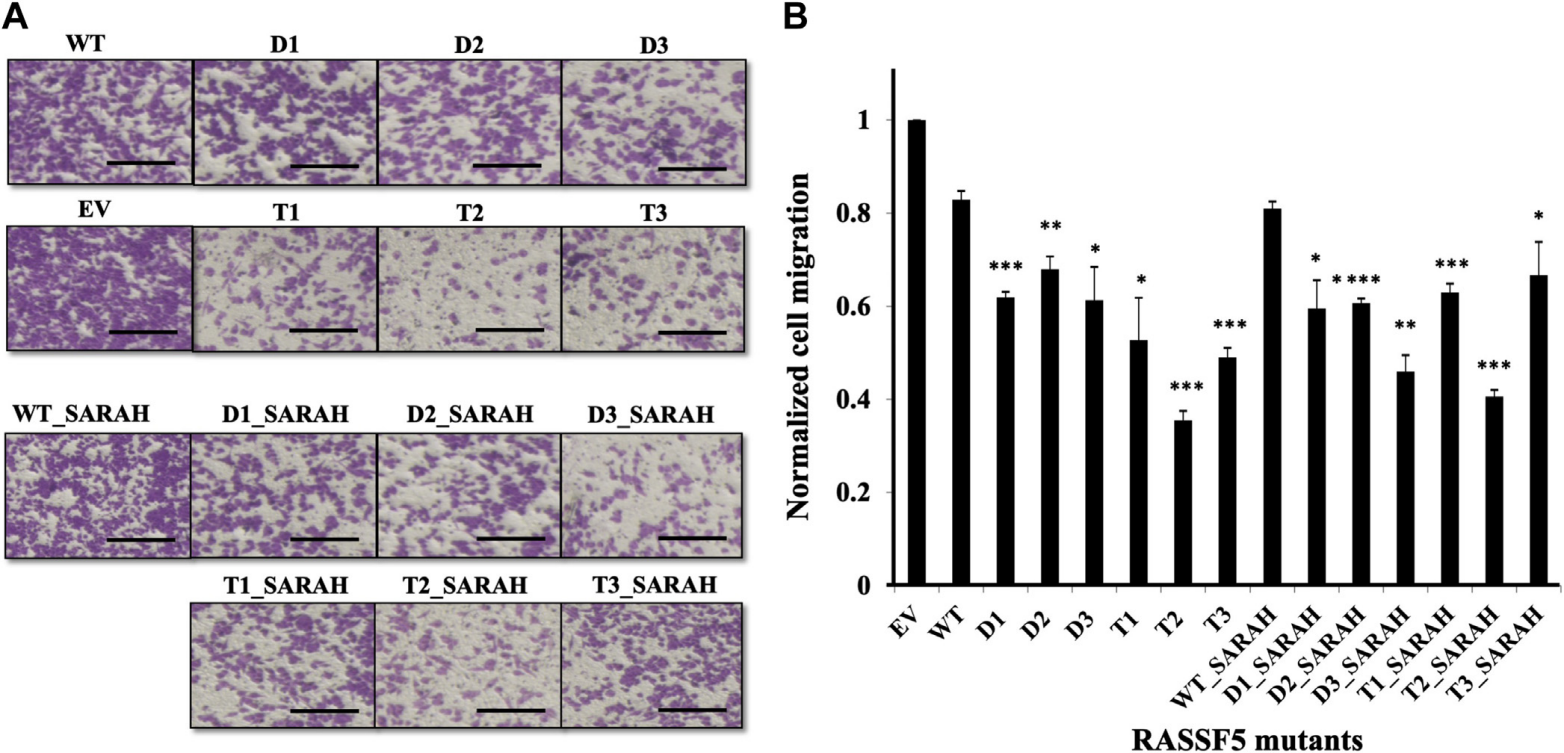
**Effects of engineered RASSF5 mutants onmigrationofA549 lung cancer cells.***A*, images of the migrated A549 cells transiently transfected with WT and engineered RASSF5 mutants either containing RA domain only (*top*) or RA+SARAH domains (*bottom*). Empty Vector (EV) was transfected as a negative control. Scale bars are 200 μm. *B*, normalized cell migration calculated as a ratio between cells transfected with RASSF5 mutant and cell transfected with EV. The results plotted represent the average of three independent experiments shown as mean ± SD. The level of significance relative to the negative control is indicated as ∗*p* < 0.05, ∗∗*p* < 0.01 and ∗∗∗*p* < 0.005 in a paired *t* test.

We next assessed the ability of RASSF5 mutants to inhibit migration of A549 cells as a proxy to metastasis (49, 50, 51, 52). For this purpose, we used the transwell assay that has been widely used for measuring motility of different types of cancer cells (53) (Fig. 6). Figure 6 shows that WT RASSF5 inhibited cell migration by about 17% compared with the negative control. All engineered RASSF5 mutants exhibited significantly higher inhibition compared with that of WT RASSF5, with the T mutants showing slightly better efficacy compared with D mutants. Once again, the T2 mutant was the most effective, exhibiting ∼64% inhibition of cell migration. Notably, addition of the SARAH domain showed no significant difference in inhibiting cell migration compared with the RA only constructs.

**Figure 7:**
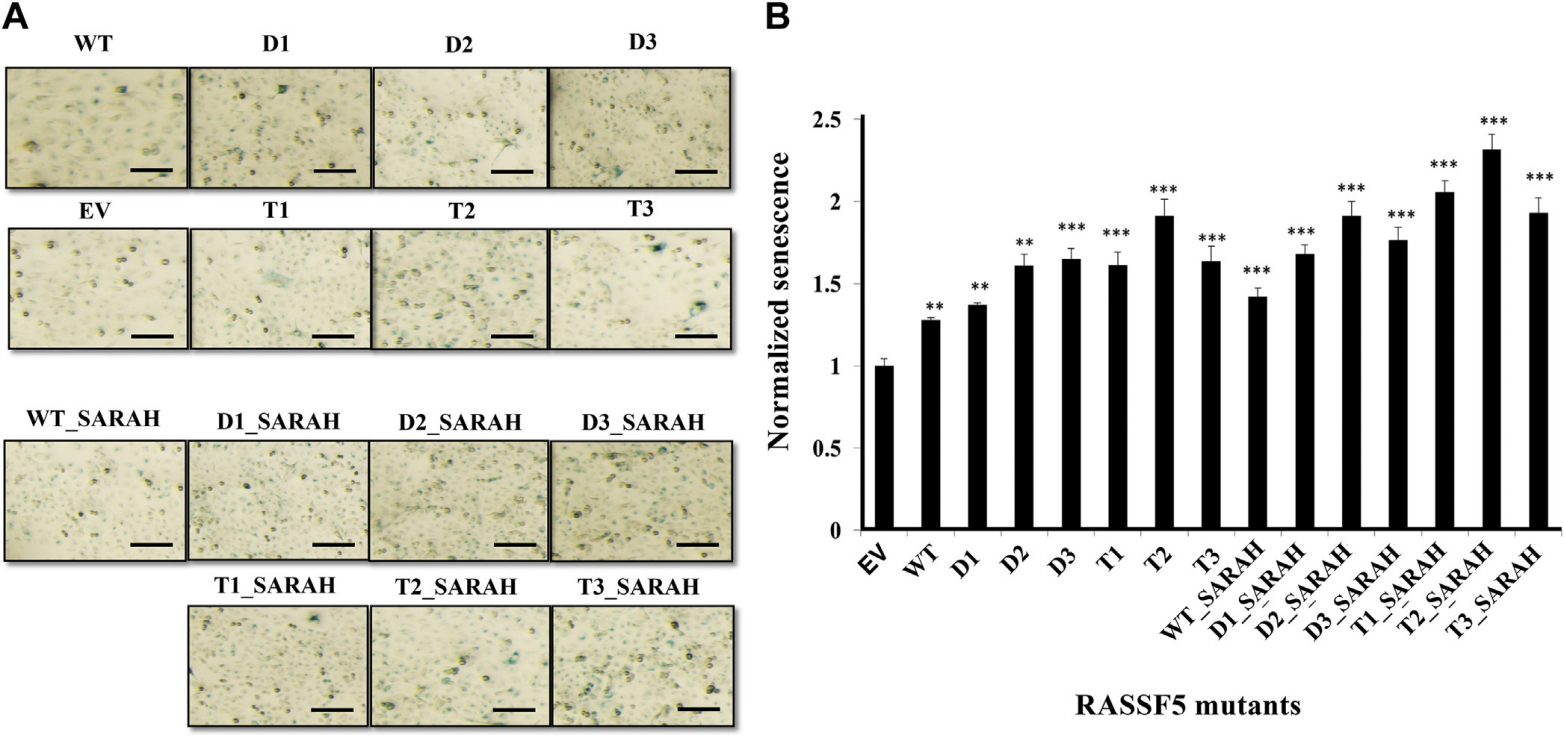
**Senescence induced by RASSF5 mutants.***A*, images of A549 cells transiently transfected with RASSF5 mutants and stained with by β-galactosidase containing either RA domains along or RA + SARAH domains. Empty Vector (EV) was used as negative control. Senescence was measured after 72 h by β-galactosidase assay. Scale bars are 100 μm. *B*, normalized senescence of RASSF5 mutants. The number of senescent cells in (*A*) was counted and normalized to that of the EV control. The results represent the average of four independent experiments shown as mean ± SD. The level of significance is indicated by ∗∗ for *p* < 0.01 and ∗∗∗ for *p* < 0.005 relative to the EV control in the paired *t* test.

Previous studies reported that RASSF5 is a potent inducer of cellular senescence (54). We hence assayed the ability of the engineered RASSF5 mutants to affect senescence of A549 cells, while using β-galactosidase activity as a marker. Figure 7 shows that introducing WT RASSF5 into cells resulted in approximately 1.2-fold increase in cellular senescence in comparison to the EV control. The engineered mutants containing only the RA domain showed 1.3- to 2-fold increase in senescence in comparison to negative control, with the highest senescence exhibited by the T2 variant. Interestingly, introduction of the SARAH domain further increased senescence by nearly 20%, for all mutants, in comparison to the RA domain only constructs. Our results thus confirmed that Ras-RASSF5 interactions are responsible for inducing cellular senescence upon interaction with Ras, and the SARAH domain plays a role in further senescence induction.

**Figure 8:**
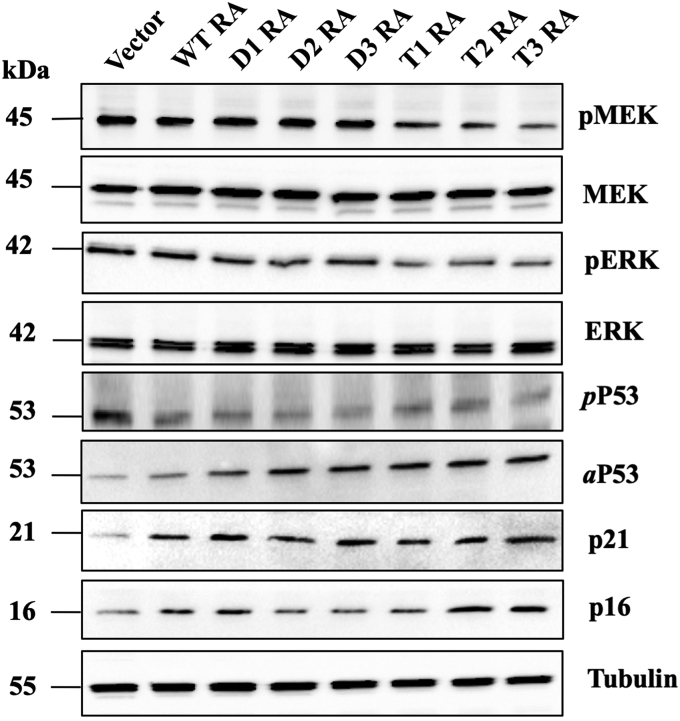
**The effect of WT RASSF5 and its mutants on downstream proteins in A549 cells.** A549 cells were transiently transfected with constructs as indicated and lysed with electrophoresis sample buffer 48 h post transfection. Lysates were analyzed by Western blotting (*WB*) for MEK phosphorylation status (*first two rows*) and ERK expression (*middle two rows*) as described under “Experimental procedures.” Same lysates were probed for p53 phosphorylation (pP53) and acetylation of p53 (aP53), and for expression of senescence markers p16 and p21 (*bottom two lanes*). Tubulin was loaded as control marker for each lane.

We have further explored how RASSF5 mutants affect function of various proteins controlled by Ras. First, we measured the change in phosphorylation of mitogen-activated protein kinase (MEK) and extracellular signal-regulated kinase (ERK), the main players in the MAPK/ERK pathway, which regulates cell proliferation through phosphorylation of many downstream targets. Figure 8 shows that transient transfection of the engineered RASSF5 mutants into A549 cells resulted in decreased in MEK and ERK phosphorylation for all T but not D mutants, consistent with the higher affinity of T mutants for the oncogenic Ras. Next, we explored the effect of RASSF5 mutants on posttranslational modifications of the tumor suppressor gene p53, since such modifications regulate the balance between prosenescence and proapoptotic pathways (55). Acetylation of p53 at Lys 382 promotes senescence by enhancing affinity to specific promoters, such as p21 (56). Phosphorylation of p53 on Ser 46, on the other hand, promotes apoptosis (57). Our data showed that in A549 cells, RASSF WT and to a greater extent RASSF5 mutants decrease p53 phosphorylation and increase its acetylation compared with negative control, thus switching the balance toward the senescence pathway (Fig. 8). Furthermore, our engineered mutants promoted enhanced expression of two well-characterized senescence markers, p21 and p16 (55, 58). We thus conclude that transient expression of RASSF5 mutants in lung cancer A549 cells stimulates cellular senescence and thus suppresses cancer.

## Discussion

Ras has been considered an undruggable oncology target due to its central role in many signaling pathways and due to the nature of its binding interface that contains no cavity where a small molecule could bind. Yet, in recent years, Ras has been targeted by several protein engineering efforts, yielding a number of high-affinity Ras binding proteins. The engineered proteins recognize either the main Ras/effector interface, a nearby site, or the Ras-dimerization interface that is important for Ras signaling (26). Several newly discovered Ras binders have been shown to counteract Ras oncogenic signaling activity by inhibiting RAS-effector interactions (59, 60), limiting aberrant cellular growth (25), inhibiting RAS-mediated signaling and transformations (23, 25), modulating downstream signaling (61) and preventing Ras-dependent tumorigenesis in both patient-derived organoids (28) and animal models (27). Thus, engineered Ras binders from different protein scaffolds present attractive candidates for pharmacological development.

Unlike previously reported Ras binders, the binder we designed utilizes a new scaffold, the Ras effector RASSF5, which interacts with the canonical effector binding site of Ras and additional residues corresponding to switch II. Hence, relative to all known effectors, the binder we examined herein possesses the largest binding interface and the most extensive van der Waals contacts with Ras (Fig. 1*A*) (7). Notably, unlike other Ras effectors, by linking Ras with other tumor suppressor genes such as p53 and retinoblastoma (Rb) protein, and by promoting senescence (40, 41), RASSF5 possesses several anticancer activities. We hypothesized that enhancing Ras-binding affinity of RASSF5 could further enhance its tumor suppressor properties by prolonging the half-life of the Ras-RASSF5 complex.

Indeed, we demonstrate that the engineered RASSF5 variants suppress various cancer-related processes in A549 human lung cancer cells that harbor a KRas mutant protein (G12S) and express no RASSF5. In particular, we show that cell viability and cell motility are decreased while senescence is increased in the presence of our RASSF5 variants. Our variants could function through two possible scenarios: (1) they could bind to Ras and compete with other Ras effectors that promote cancer, and (2) the variants could amplify the natural tumor suppressor function of RASSF5 through interactions with other proteins in tumor-suppressing pathways. In this study, we document RASSF5 mutant activities complying with both scenarios. For example, the reduction in the activity of the MEK/ERK pathway observed here is likely due to inhibition of the interaction between Ras and its direct effector, Raf, which controls the ERK-MAPK pathway. Similarly, the decrease in cell migration in the presence of RASSF5 WT and RASSF5 variants is likely due to inhibition of Ras’ interaction with PI3-Kinase. The RAS-PI3-Kinase pathway was recently implicated in regulation of cell polarity and invasion, with disruption of Ras/phosphoinositide 3-kinase interaction resulting in deactivation of Rac’s GTPase and reduction in cell motility (62).

The native tumor suppressor function of RASSF5 could explain the increase in senescence of A549 cells upon transfection with RASSF5 variants. Here, the interaction between Ras and our variants resulted in upregulation of p53 acetylation and downregulation of p53 phosphorylation, switching on the prosenescence pathway while inhibiting apoptosis. The increased senescence was also confirmed by increased expression of the senescence markers p21 and p16. Interestingly, the presence of the SARAH domain in our constructs further increases cellular senescence, indicating the importance of this domain to senescence-controlling pathways. Such an effect might be due to homodimerization of RASSF5 through the SARAH domains, or due to interactions with other, yet unknown partners. In this study, we did not investigate the effect of RASSF5 mutants on apoptosis *via* the MST1/MST2 pathway, which is also controlled by RASSF5. MST1 was shown to be downregulated in A549 cells compared with normal pulmonary epithelial cell lines (63). Hence, this pathway would not be activated even after introduction of our mutants into the cells.

It is worth noting that in all of the cellular experiments, we observed the native anticancer activity of WT RASSF5. This is not surprising since RASSF5 is not expressed in A549 cells and its ectopic expression likely translates to inhibition of oncogenic pathways, as well as to promotion of senescence. This anticancer activity is further enhanced by our engineered mutants, especially by the T mutants, in all cellular assays we performed. This suggests that enhanced affinity of the engineered RASSF5 mutants to Ras-GTP indeed results in prolonged half-life of the RAS/RASSF5 complex *in vivo*, attenuating all anticancer processed controlled by this interaction.

Since RASSF5 and other RASSF family members are inactivated by promoter hypermethylation in numerous cancer cell lines and primary cancers (32, 33), reintroduction of small proteins derived from such effectors with enhanced Ras affinity is an attractive strategy against cancer. To further develop our molecules into therapeutics, one has to overcome the problem of protein intracellular delivery. In recent years, several new strategies for protein delivery into cytoplasm have been successfully explored including utilization of cell-penetrating peptides, nanoparticles, liposomes, supercharging and bacterial toxins (64, 65). Future studies could utilize one of these delivery platforms to explore therapeutic potential of our engineered RASSF5 variants in animal models of various Ras-dependent cancers.

## Experimental procedures

### Design of the RASSF5 library for high-affinity binding to RAS and modeling of the selected mutants

First the structures of the complexes between RASSF5 RA and Ras-GTP and Ras-GDP were generated. To generate the RASSF5/RAS-GTP complex structure, we used the pdb file 3DDC as a starting point and introduced three mutations to revert the sequences of both proteins back to the WT sequences: D302K on RASSF5 and E30D/K31E on Ras using the protein design software ORBIT (66). To generate a structure of RASSF5 in complex with Ras-GDP, the structure of Ras-GDP (pdb: 2CE2) was superimposed onto the structure of the generated RASSF5/RAS-GTP complex structure and minimized. The Ras/RASSF5 interface was defined as all residues found within 4 Å from the interacting chain in the RASSF5/Ras-GTP structure. We next run the computational saturation protocol on the RASSF5/RAS-GTP complex structure. For this purpose, one binding interface position on RASSF5 was selected and mutated to all amino acids (excluding Cys, Pro, and Gly), one at a time. During the calculation, all surrounding residues were repacked and the ΔΔG_bind_ was calculated as described in our previous work (43). Based on the saturation mutagenesis results and other structural considerations described in the text, we selected seven positions for randomization in the RASSF5 library to be 218, 219, 220, 221, 234, 286, 305. We next performed two additional calculations starting from RASSF5/Ras-GTP and RASSF5/Ras-GDP complex structures, to obtain sequences compatible with high-affinity binding to Ras-GTP and Ras-GDP, respectively. For this purpose, we used the ORBIT software and redesigned the above seven positions on RASSF5 simultaneously while allowing all the neighboring residues on both proteins to repack and keep the backbone fixed. An energy function optimized for design of protein–protein interactions was used for the calculation (67). Ten thousand sequences with the lowest energy were generated for the seven designed positions in both calculations. The frequency of amino acids appearing in the designed sequences at each position was used to design the library of RASSF5 mutants. Degenerate codons were chosen to encode all desirable and the minimal number of additional amino acids.

To model how the selected RASSF5 mutants interact with Ras, we mutated the WT RASSF5 sequence to incorporate the selected mutations in the context of the RASSF5/Ras-GTP or RASSF5/Ras-GDP structures using ORBIT while allowing the neighboring residues to repack. New intermolecular interactions generated by the introduced mutations were analyzed.

### Generation of RASSF5 library

RASSF5 RA domain gene (residues 205–362) was cloned into pCTCON2 vector compatible with YSD studies. Four regions of the gene as predicted by our computational analysis were selected for mutagenesis. To increase the probability of including all mutants encoded in the library, we constructed the library using three steps all utilizing the Transfer PCR (TPCR) protocol (68). The schematic of the steps is shown on Figure S4 while all primers are summarized in Table S2. In a first step, we constructed a library of three mutants using a mixture of two mutagenic primers N2rev (encoding W, Y, and F at position 234) and a vector-complementary primer HR1fwd on top of the PCTCON2 vector containing RASSF5-RA. The library was transformed into DH5α cells, the cells were collected, and the DNA was extracted. This library served as a template for the next TPCR reaction performed with a mutagenic primer N1fwd (randomizing positions 218–221) and an RASSF5-RA complementary primer HR2rev. Again, the library was transformed into DH5α cells, the cells were collected, and the DNA extracted. This library, containing 3 × 20 × 20 × 2 × 20 = 48,000 clones, was subjected to a short PCR amplification of the fragment HR1fwd-HR2rev and the resulting double-stranded fragments were further purified. A third TPCR reaction was performed using a mixture of two mutagenic primers N3fwd (randomizing position 286 to K, R, F, Y, L, and H) and a mutagenic primer N4rev (randomizing position 305 to K, R, H, N, Q, and S) to create the second half of the library, composed of 36 mutants, and transformed into DH5α cells. From this library, the fragment HR2fwd-HR3rev was amplified in a short PCR reaction and purified. The total library thus contained 36 × 48,000 = 1,728,000 different sequences. TPCR conditions were as follows: single denaturation step (95 °C, 30 s) followed by 30 cycles of: denaturation (95 °C, 30 s), annealing (60 °C, 1 min), and elongation (72 °C, 5 min) followed by a final single elongation step of 7 min at 72 °C. All TPCR reactions were performed in a final volume of 50 μl using 0.2 ml PCR tubes. PCR reactions included the following components: 20 ng of RASSF5-pCTCON2 plasmid, 20 nM of each primer, 200 μM of each dNTP, 1× Phusion buffer, and 1.6 U of Phusion DNA polymerase (New England Biolabs, MA, USA). Short PCR conditions for gene amplification were as follows: A single denaturation step (95 °C, 1 min) followed by 29 cycles of: denaturation (95 °C, 30 s), annealing (60 °C, 1 min), and elongation (72 °C, 1.5 min) followed by a final single elongation step of 5 min at 72 °C. All PCR reactions were performed in a final volume of 50 μl using 0.2 ml PCR tubes. PCR reactions included the following components: 20 ng of RASSF5-pCTCON2 plasmid, 500 nM of each primer, 200 μM of each dNTP, 1× Phusion buffer, and 1.6 U of Phusion DNA polymerase (New England Biolabs). Single selected mutants were incorporated into pET28a vector for protein production and purification with a 6× poly-histidine at the C-terminus.

### Library transformation into yeast

To incorporate the libraries into the YSD setup, the pCTCON2 vector was linearized by digestion with NheI and BamHI (NEB). Thereafter, the two half-genes (HR1fwd- HR2rev and HR2fwd-HR3rev) were cotransformed into a competent EBY100 *Saccharomyces cerevisiae* yeast strain, with homologous recombination between the half-genes and between each half-gene and a linear pCTCON2 plasmid (Fig. S5) using a MicroPulser electroporator (Bio-Rad) as previously described by Chao *et al.* (69). The transformed yeast was grown on SDCAA plates (0.54% Na_2_HPO_4_, 0.856% Na_2_HPO_4_.H2O, 18.2% sorbitol, 1.5% agar, 2% dextrose, 0.67% yeast nitrogen base, 0.5% bacto casamino acids). Serial dilution and plating on SDCAA medium (2% dextrose, 0.67% yeast nitrogen base, 0.5% bacto casamino acids, 1.47% sodium citrate, 0.429% citric acid monohydrate, pH 4.5, and 1.5% bacto agar) were performed to determine the size of the library.

Genes corresponding to the YSD-selected RASSF5 mutants were recloned into pET28a using the TPCR protocol (68, 70). H-Ras was cloned into a pET28-Tev vector with a cleavable his-tag at the N-terminus and an AVI-tag at the C-terminal to allow for *in vivo* protein biotinylation with a BirA enzyme (71). All primers were ordered from IDT and are listed in Table S2.

### Protein expression and purification

H-Ras protein was expressed in *E. coli* in a biotinylated form, according to Tirat *et al*. (71) at 37 °C for 3 h after induction with 0.1 mM isopropyl β-d-1-thiogalactopyranoside. In this procedure, Ras with N-terminal cleavable His tag along with a C-terminal AVI-tag was coexpressed with the enzyme BirA. This enzyme recognizes the AVI-tag and covalently attaches biotin to the gene of interest. Ras was expressed in BL21(DE3) cells (NEB) and biotin (ACROS organics) was added to the growing medium at concentration of 50 μM. Biotinylated Ras was purified on a Ni-NTA column (Pierce) followed by cleavage of His-Tag using purified TEV protease. The nucleotide was further exchanged to nonhydrolyzable GTP analog (Sigma-aldrich) or GDP (Sigma-Aldrich) by incubating cleaved Ras solution with a tenfold excess of nonhydrolyzable GTP analog or GDP and 10U of Calf Intestinal Alkaline Phosphatase (Finnzymes) in the exchange buffer (Tris-HCl 20 mM, NaCl 100 mM, MgCl_2_ 5 mM, ZnCl_2_ 10 μM and (NH_4_)SO_4_ 200 mM, pH = 7.5) (72). The purity of Ras was verified by gel electrophoresis and concentration was measured by absorbance at 280 nm (Ɛ = 18,910 M^−1^ cm^−1^) and confirmed by the Bradford assay (Bio-Rad). Buffer was further exchanged using a PD-10 column (GE Healthcare) into to the Ras buffer (Tris 20 mM, NaCl 100 mM, MgCl_2_ 5 mM, pH = 7.5). Ras samples were aliquoted and stored at −80 °C.

RASSF5 WT and mutants were transformed into BL21(DE3) *E. coli* cells, induced with 1 mM isopropyl β-d-1-thiogalactopyranoside, and expressed at 15 °C overnight, purified on a Ni beads column (Adar Biotech) followed by size exclusion chromatography on a Superdex75 column (120 ml) using Ras buffer supplemented with 20 mM L-arg/Glu acid (Fig. S6). The purified mutants were further dialyzed into Ras buffer without L-arg/Glu acid. Protein purity was verified by gel electrophoresis (Fig. S7), and the concentration was measured by absorbance at 280 nm (ε = 8940 M^−1^ cm^−1^) and the Bradford assay. Protein samples were aliquoted and stored at −80 °C.

### Yeast display selection

The protocol for selection of high-affinity binders was implemented as in Chao *et al*. (69). A library of RASSF5 variants was expressed on the surface of yeast cells using the SGCAA medium (2% galactose, 0.67% yeast nitrogen base, 0.5% bacto casamino acids, 1.47% sodium citrate, 0.429% citric acid monohydrate). Galactose was added as the carbon source, to induce the GAL1 promotor, leading to overexpression of the downstream genes, AgaI, AgaII, hemagglutinin, RASSF5, and c-myc, on the surface of the yeast cells.

Yeast cells containing the RASSF5 mutant library were grown in SGCAA medium overnight. Cells were then collected and washed with 1 ml TBSA (Tris buffered saline (TBS) + 1% of bovine serum albumin (BSA)). The expression of the displayed proteins was detected by labeling the cells with 9E10 mouse anti c-myc antibody (Santa Cruz biotechnology) in a 1:50 ratio, followed by sheep anti-mouse antibody conjugated to FITC (Sigma Aldrich) in a 1:50 ratio. Binding of displayed RASSF5 to Ras was detected with 1 μM of soluble biotinylated Ras, followed by streptavidin conjugated to APC in a 1:50 dilution (Jackson immunoresearch). The labeling process was performed according to the protocol previously described by Chao *et al.* (69). The yeast cells displaying the labeled proteins were screened, analyzed, and sorted with aBD FACSAria III cell sorter (BD Biosciences). The horizontal axis provides an indication of the expression of the displayed protein (FITC fluorescence), and the vertical axis, of the binding of the presented protein to the target Ras-GTP or -GDP (APC fluorescence). The same labeling procedure was used for the RASSF5 library and for the individual selected clones, with the latter experiment being repeated four times. In the first sorting step, the 5% of the library was collected, including clones showing the highest binding and expression signal with a squared gate. During the next four rounds of sorting, triangular gates were used for collecting 2 to 5% of the highest affinity clones. In each subsequent round of selection, the concentration of Ras was lowered. Single clones were grown in SDCAA plates and later resuspended in 0.02 M NaOH in 0.2 ml plastic tubes. Tubes were incubated at 100 °C for 10 min for lysis and 1 μl of the lysate was used as template for a short PCR reaction with Taq polymerase to amplify the gene of interest (NEB). Lastly the tubes were incubated with Exonuclease I (ExoI, NEB) and Shrimp Alkaline Phosphatase (Sap, NEB) for 15 min at 37 °C to remove all primers still present in solution. Enzymes were deactivated for 15 min at 80 °C, and the samples were sent for sequencing.

### *In vitro* binding SPR measurements

Binding kinetics was performed using the OpenSPR instrument (Nicoya Lifesciences) using a Streptavidin sensor chip. All proteins were buffer exchanged into Ras buffer before the experiment supplemented with 0.1% Tween20 and 0.5% BSA. Biotinylated Ras at concentration of 50 μg/ml either in the GDP- or GTP-bound state was immobilized on the chip. Finally, various RASSF5 mutants were injected as analytes at concentration varying from 1.25 μM to 20 μM. The flow rate was maintained at 30 μl per minute, and the buffer was flowed over the chip for an additional 4 min after terminating the flow of analyte. The chip was regenerated with the solution of 10 mM HCl pH = 3, and the experiment was repeated at different analyte concentrations. SPR data was analyzed using TraceDrawer software (Nicoya Lifesciences). All *K*_D_ values were calculated using 3 to 5 concentrations per analyte.

### Tissue culture and transfections

A549 cells were kindly provided by Prof. Joel Yisraeli, (Hebrew University of Jerusalem) and grown in RPMI/10% FBS (Fetal Bovine Serum). Fourteen constructs were explored: WT RASSF5 and six mutants (T1, T2, T3, D1, D2, D3) containing only the RA domain (residues 205–362) or containing both the RA and the SARAH domain (residues 205–408) The constructs were PCR amplified and cloned into the pLEX Gateway-compatible lentiviral vector pLEX307 with a EF1a-gateway-V5 tag. Transient transfections were performed using Lipofactamine 2000 transfection reagent as described by the manufacturer (Invitrogen). Empty vector control was used as a negative control in all experiments. The transfection efficiency of the A549 cells was checked by using pEGFP-N1 (Addgene) plasmid cotransfected with individual constructs (Fig. S8).

### The MTT (3-(4,5-dimethylthiazol-2-yl)-2,5-diphenyl-2H-tetrazolium bromide) assay

For assessing cell metabolic activity, the MTT assay was performed on the transiently transfected cells (48). Briefly, the cells were incubated in 96-well plates at a density of 8 × 10^3^ cells/well with RPMI supplemented with 10% FBS. Cells were treated with 20 μl of MTT dye 37 °C for 4 h. The medium was removed and formazan crystals formed by the cells were dissolved using 150 μl of dimethyl sulfoxide (DMSO) for 10 min. The color reaction was measured at 570 nm using an enzyme immunoassay analyzer (Bio-Rad) and normalized to that of the empty vector control.

### Transwell migration assay

Cell migration was performed using Transwell chambers (8 μm pore-size, Corning Co). In this assay, after 24 h of transient transfection, cells were seeded (60,000 cells/well) on the presoaked transwell upper chambers with RPMI media. The lower compartments were filled with 650 μl of medium with 10% FBS as chemo-attractant. After incubation for 16 h, the nonmigrating cells were removed from the upper surface of the membrane by scrubbing. The cells that migrated to the lower surface of the membrane were fixed with 4% paraformaldehyde and stained with 0.4% crystal violet stain (Sigma-Aldrich Israel Ltd). The migrated cells were imaged using the cell and tissue culture inverted microscope (Olympus) and quantified using the imageJ software (73).

### Senescence assay

A549 cells were plated in a 6-well plate (Costar) and next day were transiently transfected with the RASSF5 WT and the RASSF5 mutants. At 72 h posttransfection, the cells were washed twice with PBS buffer and incubated in 1% glutaraldehyde fixing solution at room temperature for 15 min. The cells were then washed three times with PBS and were stained with 1 ml of the freshly prepared cell staining solution (Senescence Detection Kit, Abcam) at 37 °C for at least 8 h under protection from light. After staining, the cells were washed, and the senescent cells were identified as the blue-stained cells using a light microscope. At least 20 cells per field of vision were counted for each sample in five random fields to determine the percentage of the SA-β-Gal–positive cells.

### Western blotting

Cells were lysed with a lysis buffer (50 mM HEPES, pH 7.5, 10% glycerol, 150 mM NaCl, 1% Triton X-100, 1 mM EDTA, 1 mM EGTA, 10 mM NaF, and 30 mM β-glycerol phosphate). Cleared cell lysates were collected using centrifugation (12,000 rpm for 20 min) and further resolved using electrophoresis followed by transfer of the antigens to nitrocellulose membranes. The membranes were blocked with TBS-T (tris-buffered saline containing Tween-20) containing 1% low-fat milk, incubated overnight with a primary antibody, washed three times with TBS-T, incubated for 60 min with a secondary antibody linked to horseradish peroxidase (anti-rabbit AB_2307391, Jackson ImmunoResearch Laboratories, Inc), and washed once again with TBS-T. The following primary antibodies were used all purchased from Cell signaling Technology: acetyl-p53 ab (#2525), phospho-p53 ab (#2521), MEK ab (#9126), pMEK ab (#9121), p16 ab (#80772), p21 ab (#2947), ERK ab (#4695), pERK ab (#9101). Immunoreactive bands were detected using the ECL reagent (Biorad).

### Cellular assay data analysis

Data are presented as the mean ± standard deviation (SD). Comparison between WT RASSF5 and RASSF5 mutants was made by paired *t* test using the Microsoft Excel software. The level of significance was set at ∗*p* < 0.05, ∗∗*p* < 0.01, and ∗∗∗*p* < 0.005.

## Data availability

All data is included in the manuscript and Supporting information.

## Supporting information

This article contains supporting information.

## Conflict of interest

The authors declare that they have no conflict of interest with the content of this article.

## Supporting information

Figues S1–S8 and Tables S1 and S2
