# Supplementary material for: Engineered variants of the Ras effector protein RASSF5 (NORE1A) promote anticancer activities in lung adenocarcinoma
Source: J Biol Chem. 2021 Oct 27;297(6):101353. doi: 10.1016/j.jbc.2021.101353 (PMC8605244; doi:10.1016/j.jbc.2021.101353)
Supplement: Figues S1–S8 and Tables S1 and S2 [file mmc1.pdf]

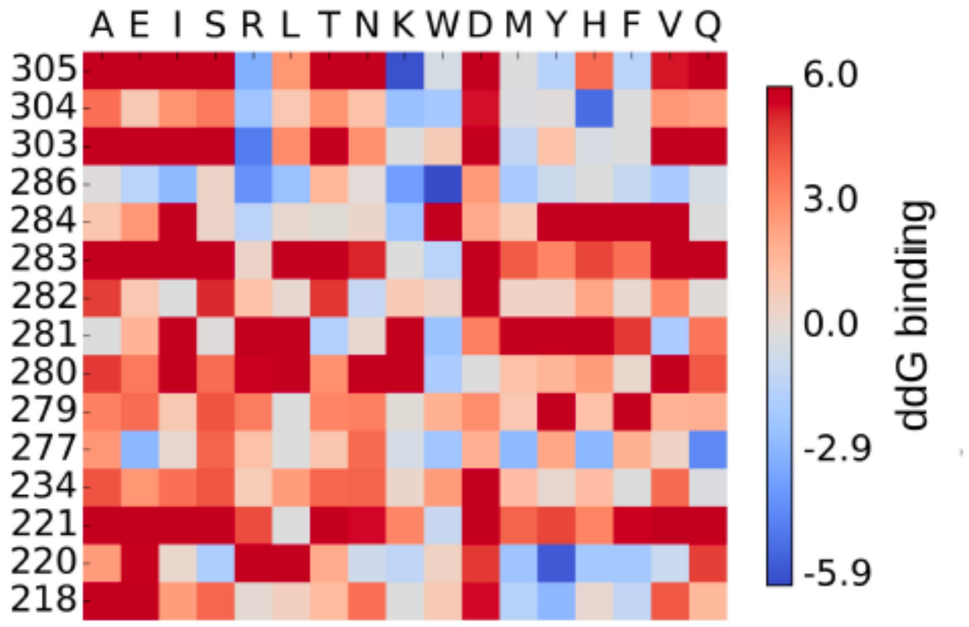

Figure S1: Computational saturation mutagenesis of RASSF5 binding interface. The Heatmap is showing calculated ΔΔGbind values for the RASSF5/Ras-GTP interaction. RASSF5 binding interface positions are shown on the left and mutations to 17 amino acids are shown on the top. One mutation is introduced at a time and E_{inter} is calculated by subtracting the energy of the single chains from that of the complex. ΔΔGbind is calculated by subtracting E_{inter} of the WT protein from that of the mutant. ΔΔGbind values are color coded from blue (stabilizing) to red (destabilizing).

Figure S2: SPR measurements for binding to Ras-GTP

WT

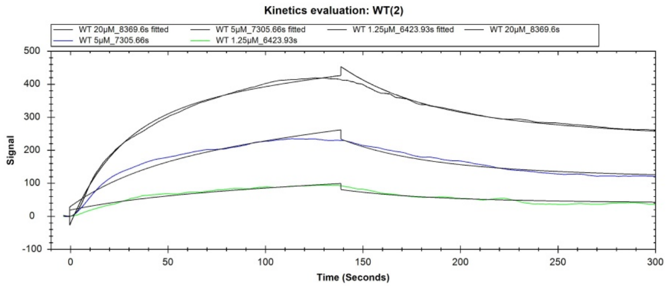

D1

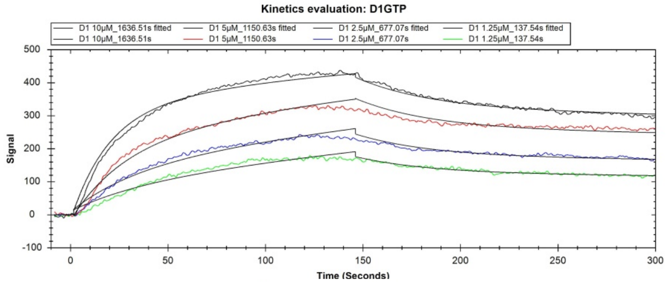

T1

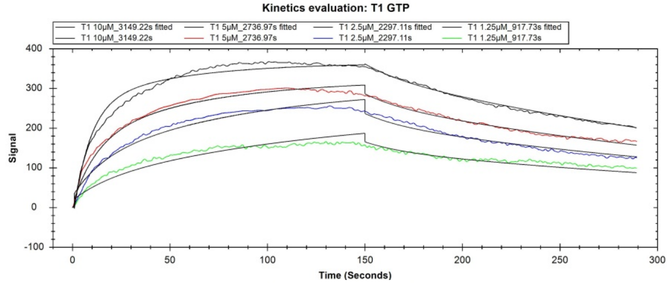

D2

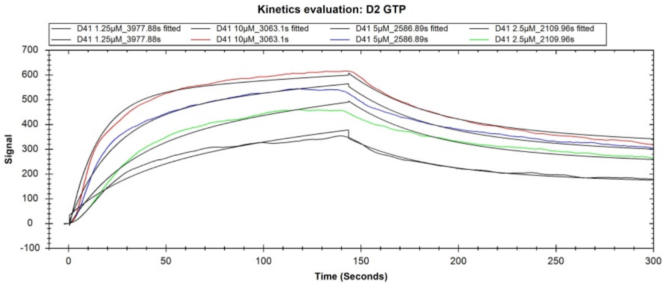

T2

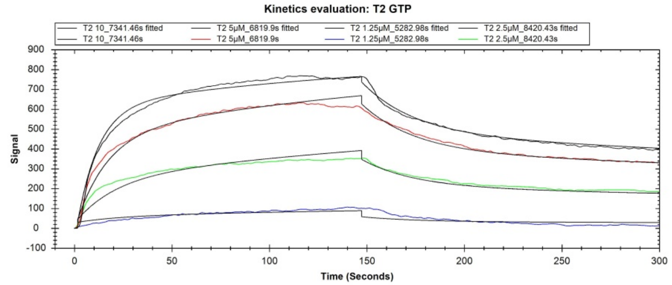

D3

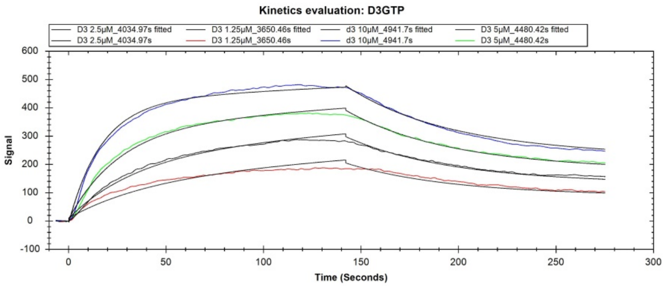

T3

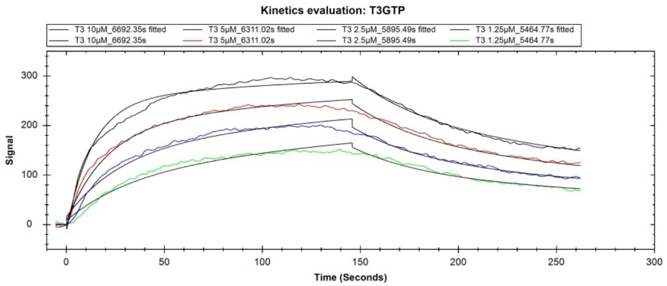

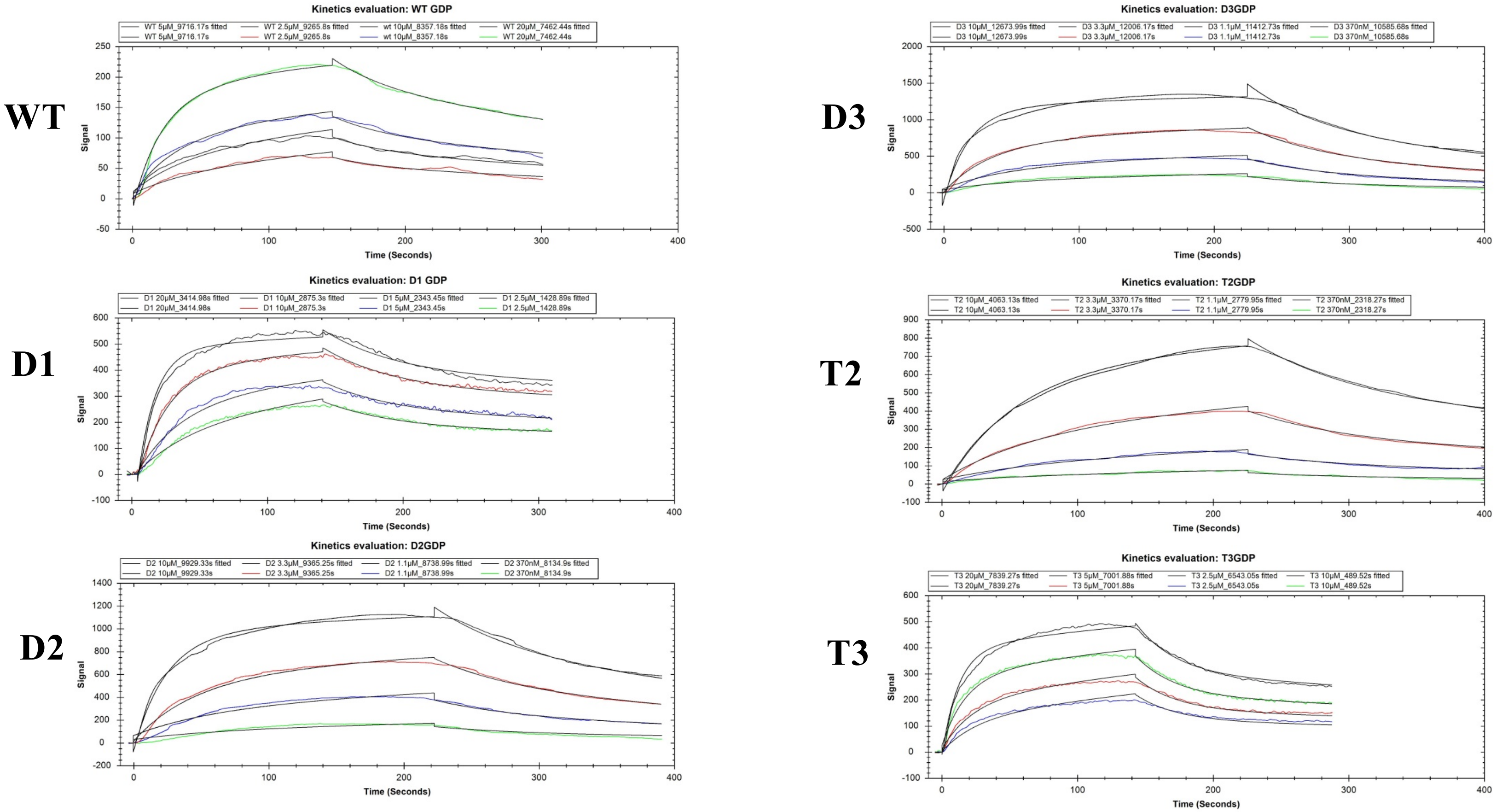

Figure S3: SPR measurements for binding to Ras-GDP

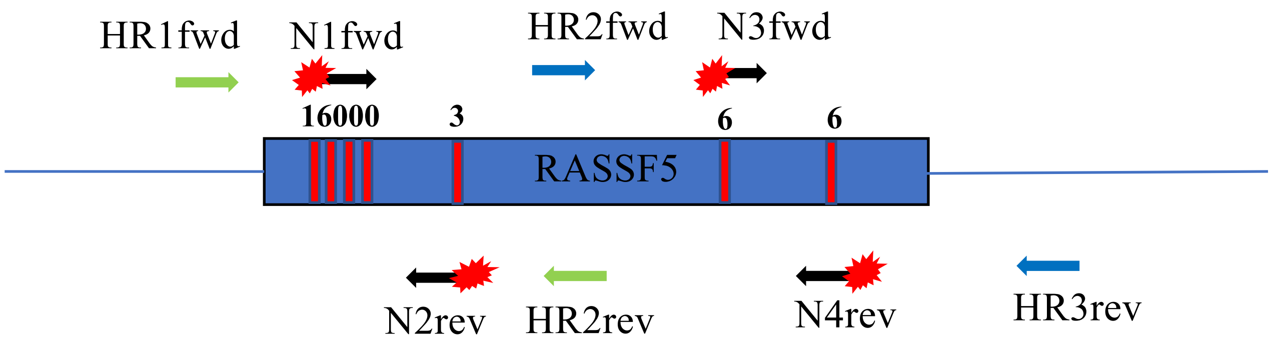

Figure S4: Construction of a focused RASSF5 library. RASSF5 (blue box) and the 4 regions where diversity was incorporated. Mutagenic primers (mutations symbolized by red explosion) were used in 4 different regions, where each vertical red line represents a designed position and numbers above represent all possible combinations of amino acids. Blue and green primers were used to amplify half-libraries (HR1fwd-HR2rev and HR2fwd-HR3rev) with an overlapping region of 150bp.

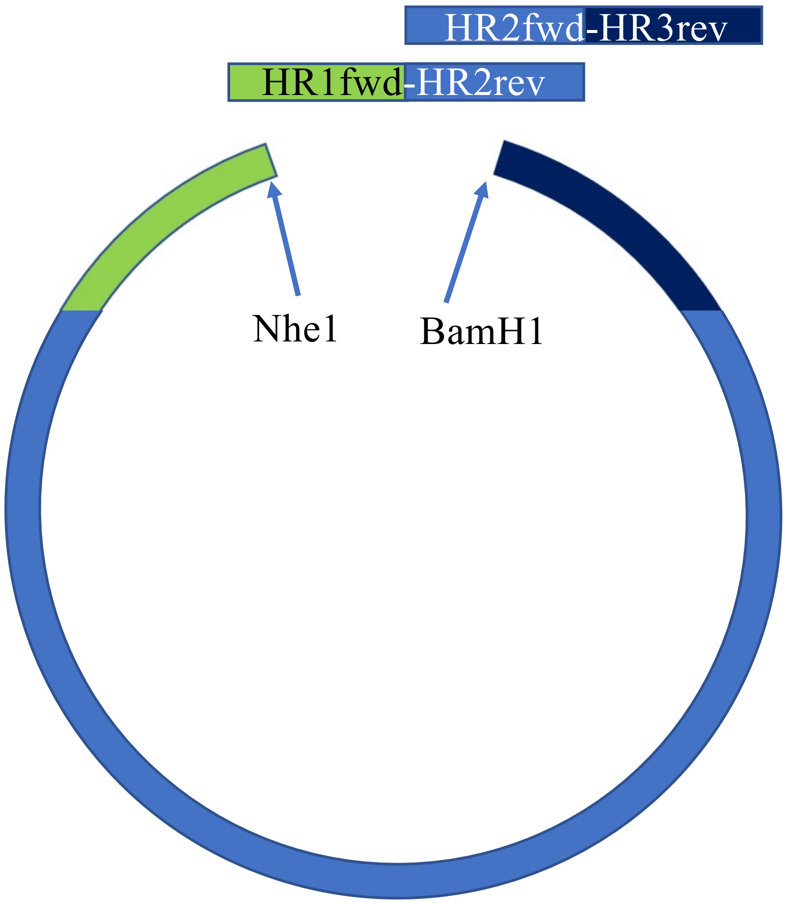

Figure S5: pCTCON2 plasmid construct for yeast transformation. Schematic representation of the linearized vector, together with the two half libraries used for transformation to yeast through homology recombination. The two PCR reactions amplified half library fragments have an overlapping region of 150bp (cyan). Each of the fragment is complementary to the linearized vector (light gray) in two separate regions (blue and green).

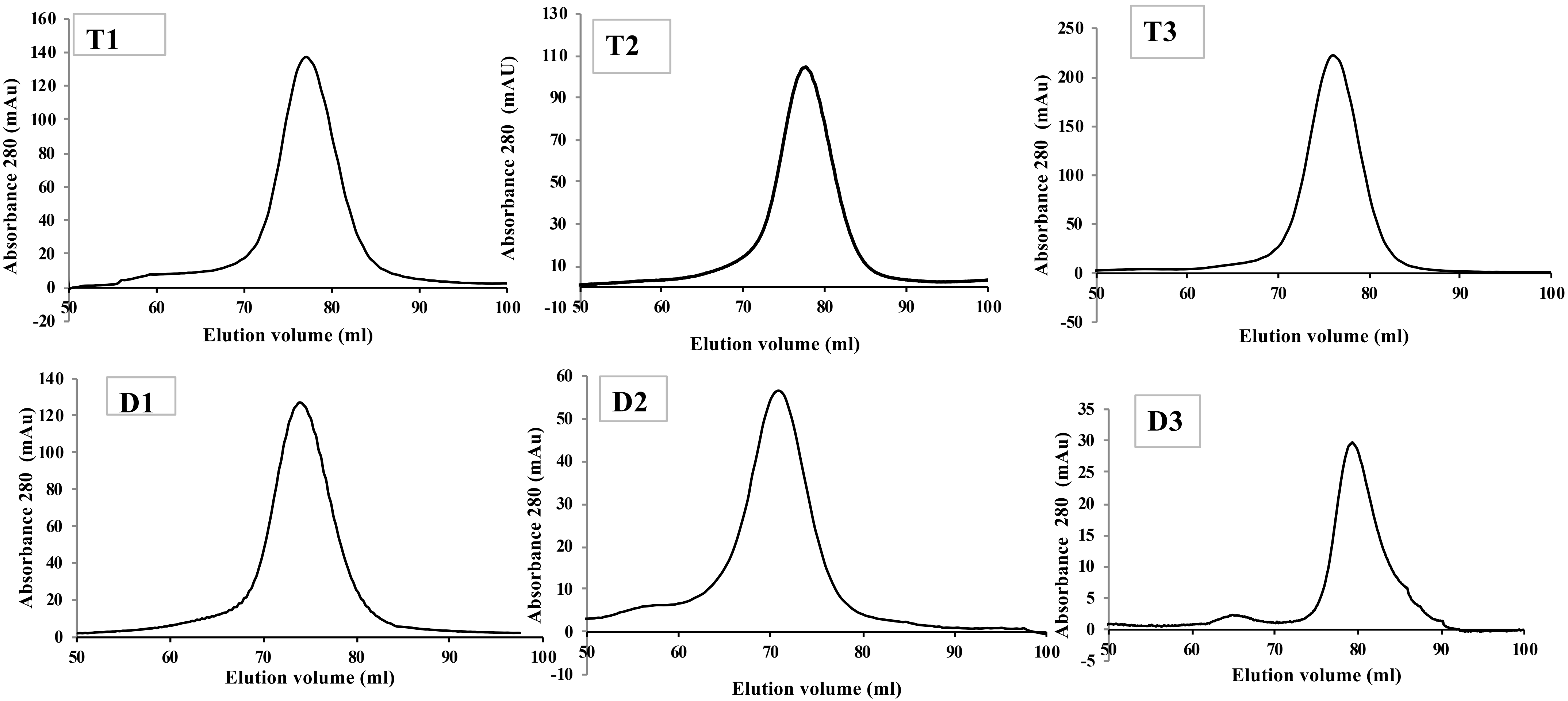

Figure S6: Purification of RASSF5 mutants by SEC. All mutants were purified in a buffer containing Tris 20 mM, NaCl 100 mM, $MgCl_2$ 5 mM, pH=7.5 supplemented with 20 mM L-Arg and 20 mM Glu. L-Arg/Glu was subsequently removed from the buffer through dialysis.

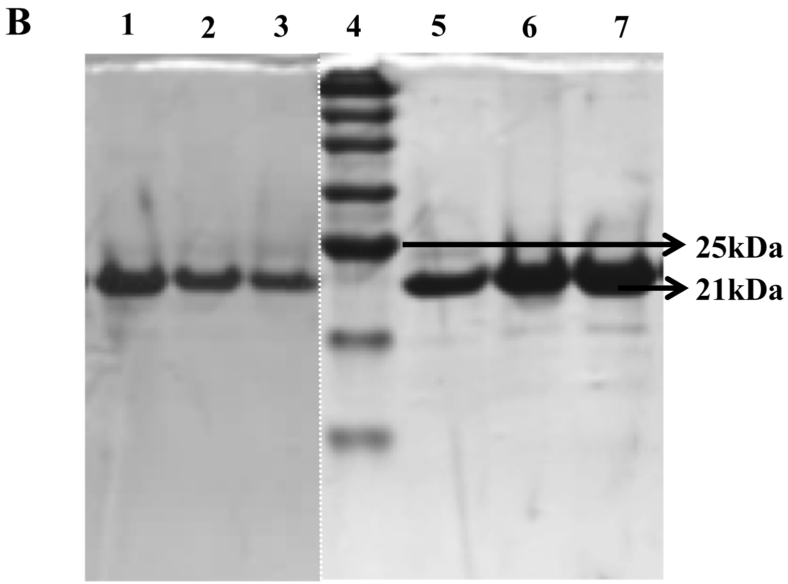

Figure S7: SDS-PAGE of purified RASSF5 mutants D1, D2, D3 (lane 1-3) and mutants T1, T2, and T3 (lanes 5-7).

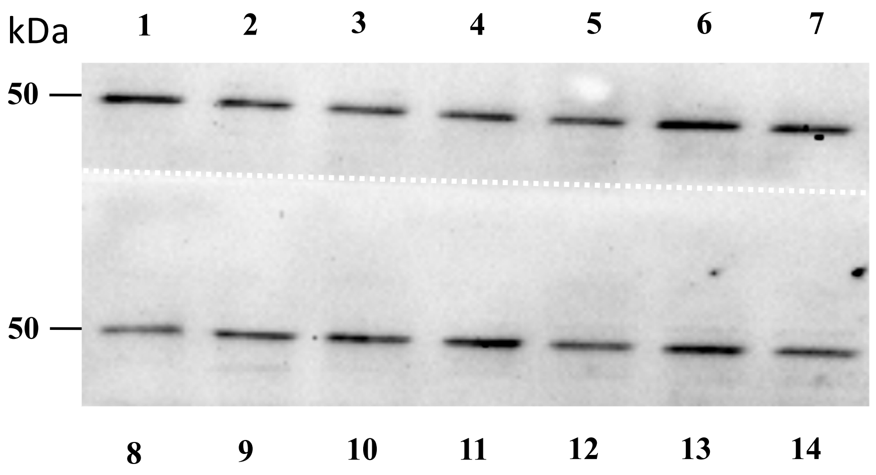

Figure S8: Co transfection of GFP plasmid with the designed constructs

A549 cells were transiently co transfected with constructs containing either RA domains alone or RA + SARAH domains (lane 8-14). and GFP plasmid as indicated and lysed with electrophoresis sample buffer 48 hours post transfection. Lysates were analyzed by Western blotting (*WB*) for GFP. RASSF5 Wild type (lane 1) and mutants D1, D2, D3 (lane 2-4), mutants T1, T2, and T3 (lanes 5-7) and RA + SARAH constructs WT (lane 8) mutants D1, D2, D3 (lane 9-11), mutants T1, T2, and T3 (lanes 12-14).

Supplementary Table S1: Sequences of mutants of RASSF5 selected to bind to Ras-GTP and Ras-GDP [a]

Ras-GTP	Ras-GDP
YRKLYRR	CCRRFKR
FRKLYKR	FRKFWHK
FRKLYKR	VLRRYHK
KHCLYKH	TRRFYRN
RYRLYKR	RRRMYRR
YRKLYKR	ATRRYRR
YRKLYRR	KHCLYRR
RWRLYRR	RWRLYRR
RFRLYRR	RCRLWRN
RFRLYRR	RYKRWHR
RFRLYRR	WRRFWHR
RLRLYKR	VRRFYRR
RLRLYKR	LRRLYRR
FWKLYRR	KHCLWHR
GWRPYKR	ARRRYHR
HRRLYRR	WRRRYHM
FRKLYKR	RMRWWRR
RFRLYRR	NIKFYKR
RLRLYKR	HCKLYKR
RLRLYKR	CCRRFKR
RLRLYKR	ASKYYKR
RFRLYRR	PSKCYRK
RLRLYKR	GTKSWRR
RFRMYRR	KHCLFRR
RWRLYRR	HSRKYRR
RTRLYKR	AWRYWRR
RYRLYKR	

[a] Only designed positions are included (218,219,220,221,234,286 and 305).

Supplementary Table S2: Primers for the library construction and cloning of RASSF5

Primer Name	Sequence
N1fwd	GATTGACAGCTATAACAGCAGGGAGNNSNNSARANNSGGCATGAAGCTGAGTGAAGATGGC
N2rev	CTTCAAATGCACTTTGATCCAACCTGTGTAGGTGCCATC+<br>CTTCAAATGCACTTTGATGSAACCTGTGTAGGTGCCATC
N3fwd	GATGCCATCAAGCAGCTAARAATCAGCAGCACCACCACG+<br>GATGCCATCAAGCAGCTAYWTATCAGCAGCACCACCACG
N4rev	GGCTGCTCAAGAAGTTCNYKGTTGTGGACAACCCACAG
HR1fwd	GCGGAGGCGGAGGGTCGG
HR2fwd	GCATTTGAAGCTCCGACGGC
HR2rev	GCTGATATGTAGCTGCTTGATG
HR3rev	GAGATCTGATAACAACAGTGTAG

Randomized or mutated positions are shown in red.
